# Genetically encoded biosensors for lignocellulose valorization

**DOI:** 10.1186/s13068-019-1585-6

**Published:** 2019-10-15

**Authors:** Guadalupe Alvarez-Gonzalez, Neil Dixon

**Affiliations:** Manchester Institute of Biotechnology (MIB), The University of Manchester, Manchester, UK

**Keywords:** Genetically encoded biosensors, Sustainable chemical production, Lignocellulose valorization, Biorefinery

## Abstract

Modern society is hugely dependent on finite oil reserves for the supply of fuels and chemicals. Moving our dependence away from these unsustainable oil-based feedstocks to renewable ones is, therefore, a critical factor towards the development of a low carbon bioeconomy. Lignin derived from biomass feedstocks offers great potential as a renewable source of aromatic compounds if methods for its effective valorization can be developed. Synthetic biology and metabolic engineering offer the potential to synergistically enable the development of cell factories with novel biosynthetic routes to valuable chemicals from these sustainable sources. Pathway design and optimization is, however, a major bottleneck due to the lack of high-throughput methods capable of screening large libraries of genetic variants and the metabolic burden associated with bioproduction. Genetically encoded biosensors can provide a solution by transducing the target metabolite concentration into detectable signals to provide high-throughput phenotypic read-outs and allow dynamic pathway regulation. The development and application of biosensors in the discovery and engineering of efficient biocatalytic processes for the degradation, conversion, and valorization of lignin are paving the way towards a sustainable and economically viable biorefinery.

## Background

Modern society is largely dependent on natural geological supplies, including crude oil, coal, and natural gas. Our reliance on these fossil fuels is, however, responsible for many causes of global concern, particularly greenhouse gas emissions and the depletion of natural resources [1, 2]. These concerns require a shift towards a sustainable economy with alternative routes to chemicals, fuels, and energy from renewable feedstocks, for which plant biomass constitutes an attractive raw material [3, 4]. During the last few decades, the concept of bioeconomy has become increasingly adopted by governments and industries, owing to its many potential environmental and economic advantages. Indeed, policies and legislations have been introduced to stimulate demand for bio-based products and biofuels derived from sustainable biomass. In the US, the bioeconomy initiative has been introduced to promote the development of new conversion technologies to upgrade biomass feedstocks [5–7]. Targets for chemical production from biomass in Europe are expected to replace at least 30% of oil-based chemicals by 2030, in accordance with the EU Bioeconomy Strategy [8].

There is a particular global socioeconomic interest in leveraging aromatic chemicals derived from lignocellulosic biomass [6, 8–10]. The major fraction of these plant aromatics is contained in the recalcitrant polymer lignin, which is found in quantities of up to 30% w/w, making it the most abundant aromatic heteropolymer on Earth [11, 12]. Thus, lignin has tremendous potential as a renewable feedstock to satisfy the current chemical and energy demands for a carbon neutral bioeconomy [10]. Lignocellulosic materials are mainly derived from non-edible residues from agriculture, dedicated energy crops and wood deposits [7, 13, 14]. Particularly high amounts of lignin are likely to be generated from the numerous cellulosic bioethanol plants which are being commissioned worldwide [15–17]. However, the current biomass utilization processes in these industries fail to recognize lignin as a valuable waste stream. Second-generation lignocellulosic biorefineries focus solely on the valorization of sugars produced from saccharification and enzymatic treatment, which are fermented to bioethanol and other biofuels, while the remaining lignin produced is used for power and heat generation [18]. Instead, efficient lignocellulosic biorefineries must strive to maximise the utilization and valorization of all the distinct biomass fractions, including lignin [19].

Enzymatic cocktails are extensively used in second-generation bioethanol production from cellulose [20]; thus, the extension of this enzymatic approach to the valorization of lignin could provide an expanded framework to a sustainable biorefinery approach. However, direct biological degradation and conversion of lignin into high value aromatic chemicals has not yet been realized at an industrial scale, owing to the intrinsic difficulties with its heterogeneity and recalcitrance. The discovery and development of efficient biocatalytic processes for the degradation, conversion, and valorization of lignin will be essential to achieve a novel, sustainable and economically viable valorization process for the long-term success of lignocellulosic biorefineries. This review focuses on the use of genetically encoded biosensors as tools to achieve these goals, and describes future avenues for technological advancement in the field. In particular, we review genetically encoded biosensors that have been developed and optimized to date to sense biomass-derived aromatic monomers and sugars towards lignin valorization applications.

## The screening bottleneck—metabolic pathway engineering and optimization

Metabolic engineering and synthetic biology present an emerging opportunity to develop direct routes to value-added chemicals from lignin. Aromatic precursors can be fed into functionally reconstructed biotransformation routes or modified to allow their introduction into de novo biosynthetic pathways. Lignin monomers can consequently be upgraded into fine chemicals such as flavouring agents, fragrances, and pharmaceuticals. Such approaches have enabled the production of numerous aromatic acids, alcohols, and aldehydes [21], and of flavonoids [22], stilbenes [23], and coumarins [24]. However, incorporation of heterologous or de novo biosynthetic pathways into a host often leads to an imbalanced metabolic flux. This imbalance can lead to toxic accumulation of intermediates that inhibit cell growth and decrease process performance. In addition, the lack of effective transport systems in these hosts can also result in reduced productivity, as these proteins are responsible for importing aromatic substrates and exporting products [25]. Pathway design and optimization for balanced enzymatic activity is, therefore, central to reach an optimal product yield and titer [26–28].

Strategies for pathway optimization and protein engineering of biocatalysts are generally based on the generation of large genetic variant libraries with the final aim of yielding highly producing strains and biocatalysts. Millions of variants are designed and obtained via rational or random mutagenesis techniques and often aided by computational predictions [29]. However, the screening and selection of target phenotypes as well as monitoring and quantifying the synthesis of chemical products constitute a major bottleneck in the design-build-test cycle, as conventional methods for metabolite measurement are low-throughput and laborious [30, 31]. Without high-throughput screening and selection methods, the ideal target strains and biocatalysts could remain unrealised. Genetically encoded biosensors provide a solution, as they can be deployed to translate the target metabolite concentration into useful detectable signals amenable to high-throughput analysis.

### Genetically encoded biosensors

In nature, cells have evolved sophisticated genetic regulatory systems to perceive changes in environmental conditions and detect intra- and extracellular metabolites [32]. In these systems, sensing of the target signal or metabolite (input) is coupled to an actuator response, which leads to the alteration of protein or gene activity within the cell (output). Such systems can be harnessed to construct genetically encoded biosensors suitable to measure the concentration of target metabolites and translate the input into a useful output (e.g., fluorescent protein and biosynthetic pathway expression). As such, biosensors provide a method to detect, monitor and regulate cellular metabolic productivity [32–35].

Several molecular systems have been used to create biosensors, including RNA riboswitches and several ligand-dependent proteins and receptors such as histidine kinases (HK) and allosteric transcription factors (aTFs) [33, 36, 37]. Extensive reviews have recently been published that cover each mechanism relating to the construction of biosensors [33, 34, 36, 38–40]. Here, we focus on protein–based systems, particularly transcriptional biosensors and their application towards biomass-derived metabolite detection.

**Fig. 1:**
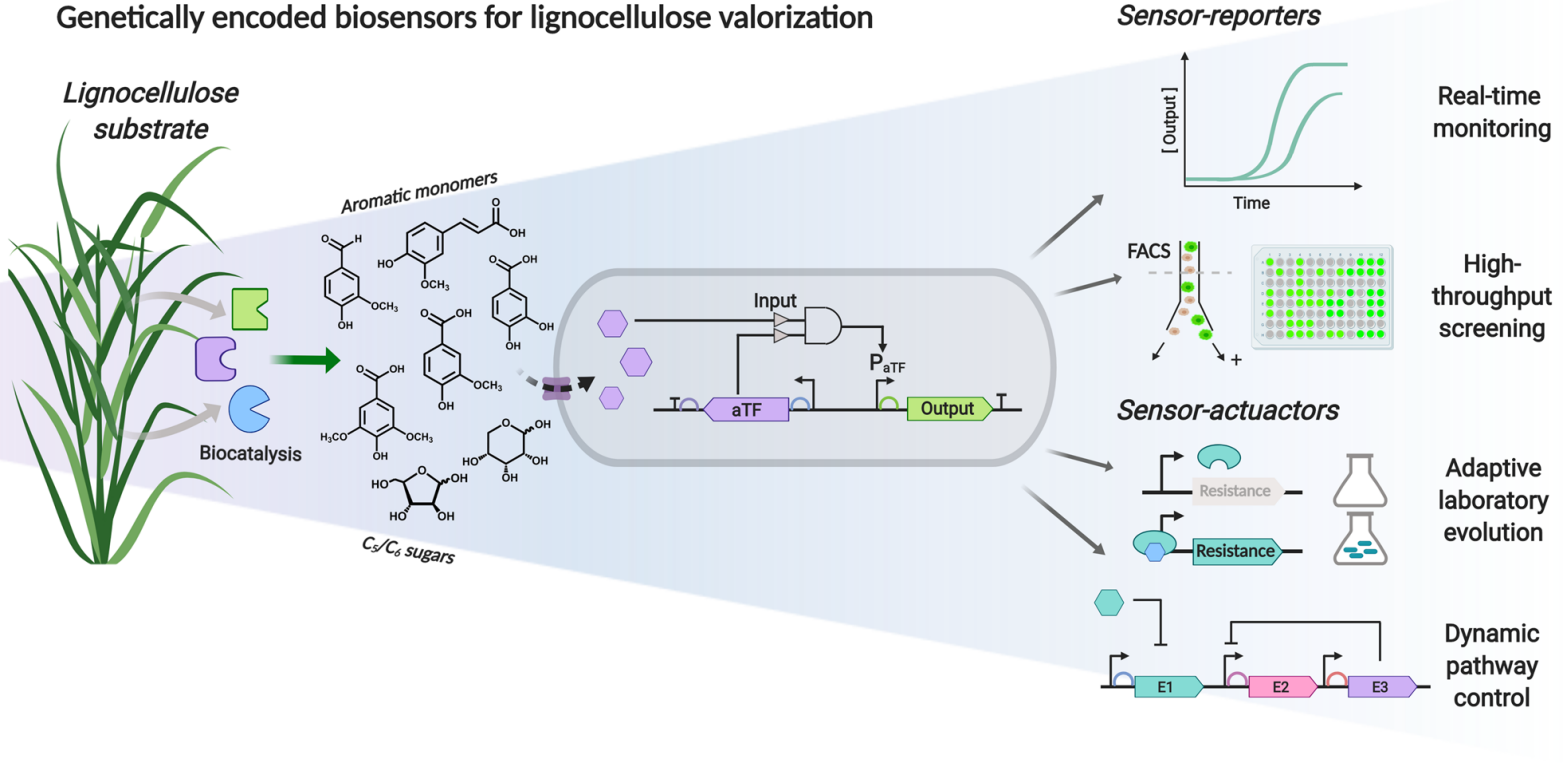
Applications of genetically encoded biosensors in lignin valorization. Transcriptional biosensors allow the coupling of aromatic monomer or sugar release detection (input) with the expression of a distinct measurable output. Reporter-based outputs can be applied in real-time metabolite monitoring and high-throughput screening of large mutant libraries using FACS. Inputs coupled to an actuator output can afford the dynamic control of enzymatic pathways according to the input level or provide an adaptive advantage under selective conditions through adaptive laboratory evolution, allowing the enrichment of high-producing strains

Transcription factors are proteins capable of regulating gene transcription by binding to specific DNA operator sequences within promoters, in turn controlling RNA polymerase activity. These regulators may activate (activators) or repress (repressors) the expression of actuator genes and regulators [41]. Allosteric TFs (aTFs) undergo a change in their conformation when activated by an effector molecule, thus altering their DNA-binding activity [42]. Allosteric transcriptional regulation has become a particularly popular method for metabolite sensing due to the myriad of native effector molecule specificities [33] including numerous aromatic compounds (Table 1), and have been constructed as tools for distinct applications and outputs (Fig. 1).

**Table 1:** Genetically encoded biosensors developed and applied to sense aromatic monomers and sugars towards lignin valorization

Target molecule(s)	Sensing element	Output element	Host organism	References
Aromatic monomers				
Protocatechuic acid	PcaU	Engineered P_pcaU_, P_3B5B_	*Escherichia coli*	[69, 101]
	PcaU	Engineered P_pcaU_	*Pseudomonas putida*	[70]
	PobR variant	P_pob_	*Escherichia coli*	[75]
	PcaV	Engineered P_PV_	*Escherichia coli*	[76]
Vanillin	EmrR	P_emrR_	*Escherichia coli*	[68]
	Yap1/Msn2	P_adh7_	*Escherichia coli*	[89]
	QacR variant	P_qacA_	*Escherichia coli*	[74]
	*nd*	P_yeiW_	*Escherichia coli*	[64]
	EmrR	Engineered P_emrR_	*Escherichia coli*	[67]
	EmrR	P_emrR_	*Escherichia coli*	[79]
	YqhC	P_yqhD_	*Escherichia coli*	[102]
	PcaV variant	Engineered P_PV_	*Escherichia coli*	[76]
	VanR	Engineered P_VanO_	*Escherichia coli*	[103]
	*nd*	P_LPD00563_	*Rhodococcus opacus*	[104]
Vanillic acid	VanR	P_vanCC_	*Escherichia coli*	[101]
	EmrR	P_emrR_	*Escherichia coli*	[67]
	EmrR	P_emrR_	*Escherichia coli*	[79]
*p*-Coumaric acid	EmrR	Engineered P_emrR_	*Escherichia coli*	[67]
	EmrR	P_emrR_	*Escherichia coli*	[79]
	PadR	P_padC_	*Corynebacterium glutamicum*	[48]
Ferulic acid and related compounds	FerC	Engineered P_LC_ and P_PC_	*Escherichia coli*	[66]
	FerC	P_ech_	*Escherichia coli*	[88]
Cinnamaldehyde	BldR	P_Sso2536adh_	*Escherichia coli*	[105]
Benzoic acid	EmrR	P_emrR_	*Escherichia coli*	[79]
	NahR	P_sal_	*Escherichia coli*	[85]
	BenR	P_benA_	*Escherichia coli*	[80]
	*nd*	P_LPD06580_	*Rhodococcus opacus*	[104]
4-Hydroxybenzoic acid	EmrR	P_emrR_	*Escherichia coli*	[79]
	PobR variant	P_pob_	*Escherichia coli, Pseudomonas putida*	[75, 86]
	*nd*	P_LPD06764_	*Rhodococcus opacus*	[104]
2-Hydroxybenzoic acid	NahR	P_sal_	*Escherichia coli*	[85]
Benzaldehyde	BldR	P_Sso2536adh_	*Escherichia coli*	[105]
Benzoic acid derivatives	XylS	P_m_	*Escherichia coli*	[71]
Salicylic acid	EmrR	P_emrR_	*Escherichia coli*	[79]
	Engineered AraC	P_BAD_	*Escherichia coli*	[106]
Salicylaldehyde	BldR	P_Sso2536adh_	*Escherichia coli*	[105]
	NahR	P_sal_	*Escherichia coli*	[85]
Salicylic acid derivatives	XylS, NahR	P_m_, P_sal_	*Escherichia coli*	[71]
Phenol	EmrR	P_emrR_	*Escherichia coli*	[79]
Phenol derivatives	DmpR	P_o_	*Escherichia coli*	[71]
Syringaldehyde	EmrR	P_emrR_	*Escherichia coli*	[68]
BTX	XylR	Engineered P_o’_	*Escherichia coli*	[65]
Other aromatic aldehydes	BldR	P_Sso2536adh_	*Escherichia coli*	[105]
	PcaV variant	Engineered P_PV_	*Escherichia coli*	[76]
C5/C6 sugars				
Xylose	XBP−XynA	P_xylF_	*Escherichia coli*	[107]
	XylR	Engineered P_GAL1/XylR_	*Saccharomyces cerevisiae*	[91]
	XylR–XBP	P_xylF_	*Escherichia coli*	[95]
d-Arabinose	araF	FRET	*Escherichia coli*	[94]
	AraC variant	P_BAD_	*Escherichia coli*	[90]
l-Rhamnose	RhaS	P_rhaBAD_	*Escherichia coli*	[96]
l-Mannose	RhaS	P_rhaBAD_	*Escherichia coli*	[96]
Cellulase	CelR	P_TRC_	*Escherichia coli*	[92]
Maltose	AraF	FRET	*Escherichia coli*	[94]
Glucose, glutamate	MglB, YbeJ	FRET	*Escherichia coli*	[93]

*nd* not determined

#### Sensor reporters—biosensors for phenotype screening

Genetically encoded biosensors are well suited for phenotype screening when the detection of a target metabolite is coupled to fluorescent protein expression [36]. These systems generate an output signal that is proportional to the final product yield, allowing the biosensor to be used in applications such as the real-time monitoring of metabolite bioproduction (Fig. 1). Owing to the correlation between fluorescence output and enzyme or pathway efficiency, substrate utilization and final product production can be monitored to facilitate optimization [43].

When coupled to sorting techniques such as fluorescence-activated cell sorting (FACS) or microfluidics, biosensors permit the high-throughput screening and sorting of large mutant genetic libraries for the selection of high-producing phenotypes and biocatalysts, useful in directed evolution applications (Fig. 1) [27, 35]. When assessing variant libraries, conventional screening techniques such as high-performance liquid chromatography (HPLC) or mass spectrometry (MS) are restricted to only 10^3^ clones per day, and are time-consuming and laborious. In contrast, biosensors and FACS-mediated screening can enable the high-throughput screening of up to 10^7^ clones per day [44]. Transcriptional biosensors have been particularly successful in the sorting and selection of improved or novel enzymes and pathways using FACS [31, 33, 43, 45]. For instance, a biosensor based on the dimethyl phenol regulatory protein (DmpR) was utilized in the high-throughput screening of 6 × 10^6^ mutants from a tryptophan-indole lyase library to select improved enzymes using this sorting technique [44]. Other genetically encoded biosensors have also been employed for the analysis of metabolic production of mevalonate [46], lysine [43], and triacetic acid lactones [47]. In addition, when biosensors are coupled to microfluidics, library sizes of more than 10^8^ clones can be screened per day [48]. In general, biosensor-based techniques provide optimal sensitivity and high performance, closing the gap between variant generation and the subsequent selection from these libraries.

#### Sensor actuators—biosensors for dynamic pathway control and adaptive evolution

Besides providing information about the intracellular presence of target metabolites, biosensors can alternatively be employed as actuators when effector binding is coupled to expression of genes encoding enzymes with activity towards the effector (Fig. 1). Heterologous bioproduction can lead to toxic intermediate accumulation and metabolic stress which retards cell growth, rendering the process ineffective (see “The screening bottleneck—metabolic pathway engineering and optimization” section) [26]. Utilizing biosensors as regulatory systems for dynamic pathway control can alleviate these problems by allowing temporal and on-demand gene and pathway expression, reducing the metabolic burden of toxic intermediates and improving the efficient utilisation of cellular energy and resources [28]. For instance, Zhang et al. [49] developed a sensor-actuator system based on the fatty acid-responsive FadR aTF, which allowed cells to sense sufficient accumulation of the intermediate fatty acyl-CoA and regulate a pathway for its conversion into fatty acid ethyl ester. This strategy achieved balanced cell growth rate and increased biodiesel production by threefold [49]. Similarly, Xu et al. [50] constructed a sensor-actuator system based on the malonyl-CoA responsive FapR aTF to dynamically regulate a fatty acid (FA) biosynthesis pathway upon malonyl-CoA levels. In this way, the system achieved a balanced metabolism with a 2.1-fold increase in FA production compared to the uncontrolled pathway [50, 51].

Moreover, controlling evolution with biosensors by linking sensing and cell fitness (i.e., through regulation of an antibiotic resistance gene) enables the enrichment of mutant libraries with improved phenotypes and productivities through adaptive laboratory evolution (ALE). Adaptive evolution approaches have proven a valuable tool to improve enzymatic activity and metabolic productivity under desired selective conditions [52]. This approach has been demonstrated for the increased production of numerous metabolites, including tyrosine [53], l-valine [54] and muconic acid [55]. This approach also allows control of cell-to-cell variation in metabolic engineering applications, allowing the fine-tuning of synthetic pathways for optimized production [56, 57]. Xiao et al. [58] developed a method to exploit cell-to-cell variation for increased biosynthesis of free fatty acid (FFA) and tyrosine. The method is based on the coupling of an FFA biosensor with the expression of a tetracycline resistance gene, allowing the continuous enrichment of high-producing strains under tetracycline selective conditions. As a result, the approach achieved a threefold increase in FFA and tyrosine production [58].

## Development of biosensors for lignin-derived monomers

Owing to the distinct genetic configurations and applicability of genetically encoded biosensors, these systems constitute ideal technologies for lignocellulose valorization. To date, numerous biosensors have been developed to sense aromatic monomers, including phenolic compounds obtained through direct lignin depolymerisation and other potential intermediates relevant to biomass valorization (Table 1). The variety of developed aromatic monomer biosensors provide the opportunity to facilitate the regulation, evolution, and selection of highly productive biocatalysts, pathways, and strains useful in lignocellulose degradation and valorization through diverse biosensor-based applications.

### Development and optimization of biosensors based on available regulatory systems

The majority of the aromatic-responsive biosensors developed to date are based on the adaptation of characterized regulatory elements from aromatic degradation pathways and tolerance systems from soil microorganisms. These elements predominately consist of aromatic-responsive transcription factors and their cognate promoters/operators [59–61], and have been prominently derived from *Pseudomonas* spp., *Acinetobacter* spp. and the well-characterized lignin-degrading *Sphingobium* sp. strain SYK-6. Other species have also been identified as efficient lignin-degrading microorganisms, such as *Rhodococcus jostii* RHA1 [60, 62, 63] that could thus constitute an ideal host and source of responsive elements for the development of biosensors. The discovery and characterization of these aromatic-responsive elements have been partly accomplished by different strategies involving gene regulation in response to the target aromatic. For instance, Sana et al. [64] utilized an RNA-seq analysis of *E. coli* cultures treated with vanillin to identify upregulated genes. The identified P_yeiW_ promoter upstream from these genes was thus used to construct a vanillin biosensor by placing it upstream of a reporter gene.

**Fig. 2:**
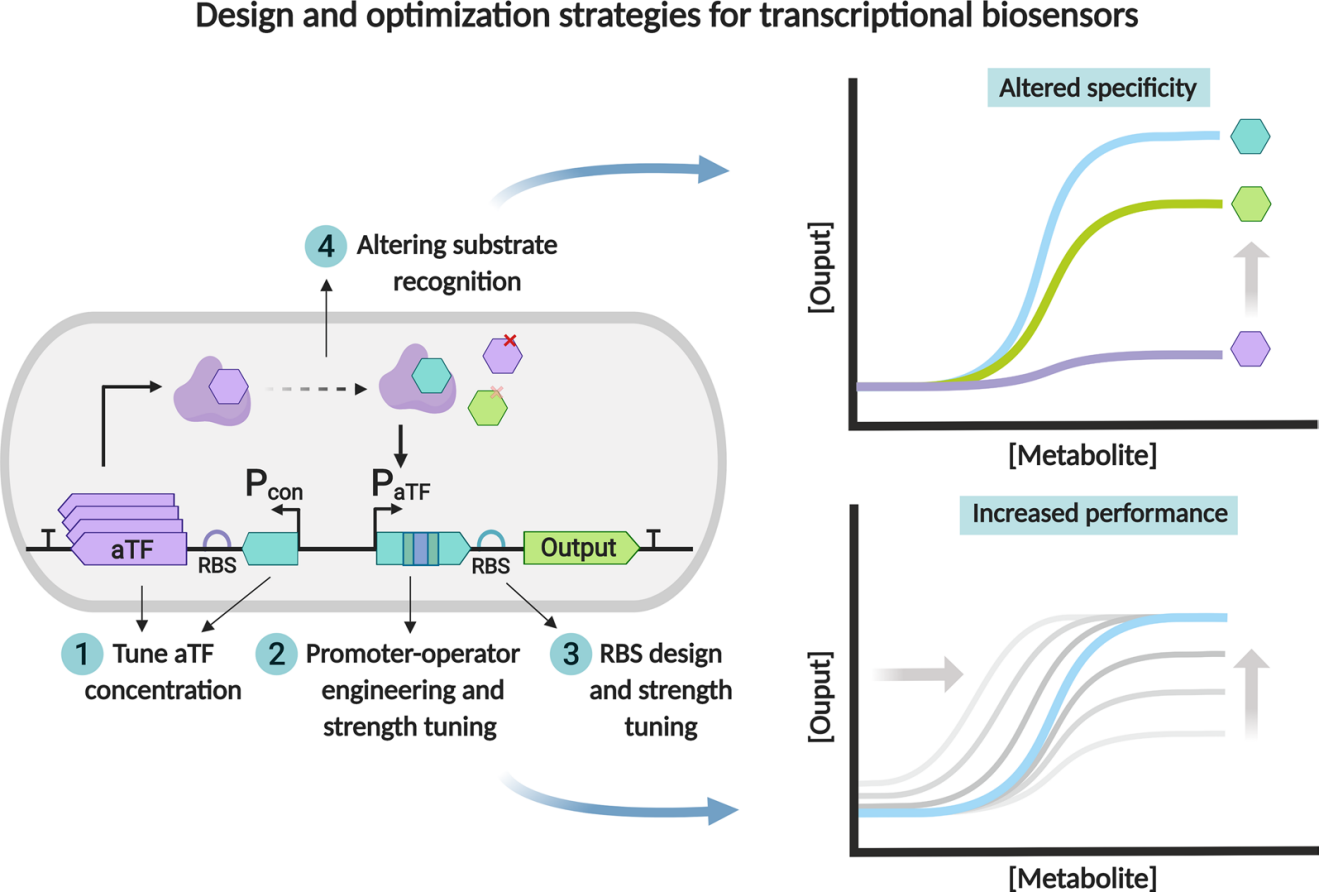
Design and optimization strategies for transcriptional biosensors. The performance of transcriptional biosensors can be altered by fine-tuning the expression levels of the allosteric transcription factor (1), engineering the cognate promoter and operator sequences (2), and/or ribosome binding site (3). The biosensor substrate recognition scope can be altered by engineering the specificity of the transcription factor (4)

Once the responsive genetic elements have been identified, fine-tuning is often required to obtain optimal performance. Different strategies have been utilized in the design and optimization of biosensors, predominantly targeting different genetic elements affecting the dose–response curve (Fig. 2). A common strategy has been based on the engineering of the cognate promoter sequences and operator sites that bind to the aromatic-responsive aTFs (Fig. 2) [38]. Kim et al. [65] initially described an optimized BTX-responsive biosensor built from the previously characterized xylose regulator (XylR) aTF from *Pseudomonas* spp., where the cognate promoter was first upstream from the luciferase reporter gene, and then was exchanged with an engineered promoter of higher strength that lacked certain XylR upstream activating sequences. The exchange resulted in increased fold change of more than 3000-fold in response to o-Xylene [65].

The binding of the aTF to its cognate promoter sequence can also be rationally modulated by developing chimeric promoter–operator sequences. Machado and Dixon [66] developed a biosensor based on the FerC repressor, a MarR-type regulator protein and its corresponding transcription factor binding (operator) site from *Sphingobium* sp. SYK-6. The reporter output signal was amplified by engineering chimeric promoter–operators, composed of the native operator sites and phage-derived promoter elements. The developed biosensor afforded detection of various hydroxycinnamic acids, including ferulic acid and *p*-coumaric acid with more than 20-fold response [66]. A similar hybrid promoter engineering approach was also recently used to develop promoters of higher strength induced by phenolic compounds present in lignocellulosic hydrolysates, including vanillin and vanillic acid. The strategy was based on the reconstruction of the spacer sequence of the native phenolic-inducible P_emrR_ promoter from *Escherichia coli*, which resulted in up to 4.6- and 9.2-fold increase in output signal in response to vanillin and vanillic acid, respectively [67]. These engineered promoters and their corresponding aTFs will thus be useful towards the development of biosensors for lignin valorization applications.

An alternative approach employed to enhance biosensor performance has been to adjust the intracellular concentration of the aromatic-responsive aTF (Fig. 2). Ho et al. [68] utilized a library of constitutive promoters of varying strength to regulate the expression of the vanillin responsive EmrR aTF. Increased aTF concentration with optimal promoter strength permitted an improved performance in response to vanillin and a broader specificity range towards other aromatics [68]. In addition, tuning and development of aromatic transcriptional biosensors by ribosome-binding site (RBS) selection and modification have been used to improve biosensor performance (Fig. 2). Siedler et al. [48] developed a *p*-coumaric acid-responsive biosensor based on the PadR aTF. The initial biosensor design resulted in the overexpression of PadR, which led to the inhibition of its cognate promoter. Modifying the downstream RBS, reduced aTF expression and consequently optimized fold change (130-fold) in response to *p*-coumaric acid was achieved [48].

Biosensors have also been optimized by targeting both the promoter–operator sequences and their RBS. A protocatechuic acid (PCA) biosensor was developed in *E. coli* based on the characterized PcaU aTF from *Acinetobacter*, an IcIR-type TF involved in the catabolism of PCA [69]. Combined engineering of the PcaU-responsive promoter sequence and the downstream RBS afforded an increase in reporter output signal and improved fold change (up to 14-fold) in response to PCA [69]. In follow-up studies, the PcaU-derived biosensor was transferred into *Pseudomonas putida,* achieved by promoter engineering and protein directed evolution. These approaches allowed the optimization of aTF–DNA interaction and aTF dimerization, which ultimately afforded a > 12-fold output signal at 30 mM PCA concentration [70].

Xue et al. [71] developed and optimized a set of biosensors based on an array of previously identified transcription factors and cognate promoter sequences from *Pseudomonas* (DmpR, HbpR, NahR, and XylS) [71]. These biosensors jointly responded to 20 different aromatic compounds, some of which can be derived as products of lignocellulose degradation and/or valorization, including several benzoate, salicylate, and phenol derivatives [71]. In these biosensors, the expression of the aTF was fine-tuned using a constitutive promoter library, and expression of reporter gene was optimized by RBS engineering [71].

These examples provide an indication of the promising array of lignocellulose-derived substrates and intermediates that can be detected using available genetically encoded biosensors (Table 1). The development of additional and novel substrate specificities would, however, be useful to increase the scope of lignocellulose-derived products that can be detected and to, therefore, enhance the utility of genetically encoded biosensors.

### Design and selection of aromatic-responsive biosensors with altered substrate recognition

Protein engineering and directed evolution techniques constitute valuable approaches to extend the effector recognition capabilities of transcriptional biosensors, which is useful for the detection of molecules, where natural biosensor systems have yet to be discovered or characterized (Fig. 2) [42, 72]. In particular, unique aromatic-responsive parts with novel metabolite specificities have been engineered through random and site-directed mutagenesis techniques or computational design. Computational methods offer investigation of wider mutagenic arrays, supporting the de novo design of customized specificities within particular protein structures, such as ligand and DNA-binding domains [42, 73]. De los Santos et al. [74] utilized a rational, computational structure-based homology modelling approach to develop a library of QacR variants, a transcriptional repressor from the TetR family, which resulted in the selection of variants with altered specificity towards the monomeric aromatic vanillin [74]. Jha et al. [75] engineered novel specificity towards PCA through the Rosetta homology-based redesign of the ligand-binding domain of the PobR transcription factor, which is natively responsive to 4-hydroxybenzoic acid (4HB). This modelling approach allowed for the in silico prediction of an aTF variant library from a homology model. FACS screening was then utilized to select improved clones, yielding a variant aTF with high transcriptional induction in response to PCA and increased performance with the native 4HB inducer [75]. More recently, the MarR-type repressor pcaV, which is natively responsive to PCA, was reengineered using a biosensor-based FACS selection method to alter its substrate specificity. The directed evolution approach resulted in the identification of a pcaV variant (Van2) that permitted the in vivo detection of vanillin and other aromatic aldehydes, and was used to construct a vanillin biosensor [76].

## Application of biosensors in lignocellulose valorization

### Functional metagenomic screening

Release of unmodified aromatic monomers from lignin following harsh chemical pre-treatment has proven challenging [77]. In contrast, bacterial lignolytic activities can afford the release of unmodified low molecular weight aromatics from lignin-rich waste streams. Several bacterial enzymes are known to degrade and transform lignocellulosic biomass into monomeric units [78]. Due to the heterogeneous and highly cross-linked nature of lignin, no enzymatic process has been demonstrated to achieve this conversion with high levels of efficiency. As such, further enzyme development and discovery would be a valuable step towards an integrated lignocellulose biorefinery.

**Fig. 3:**
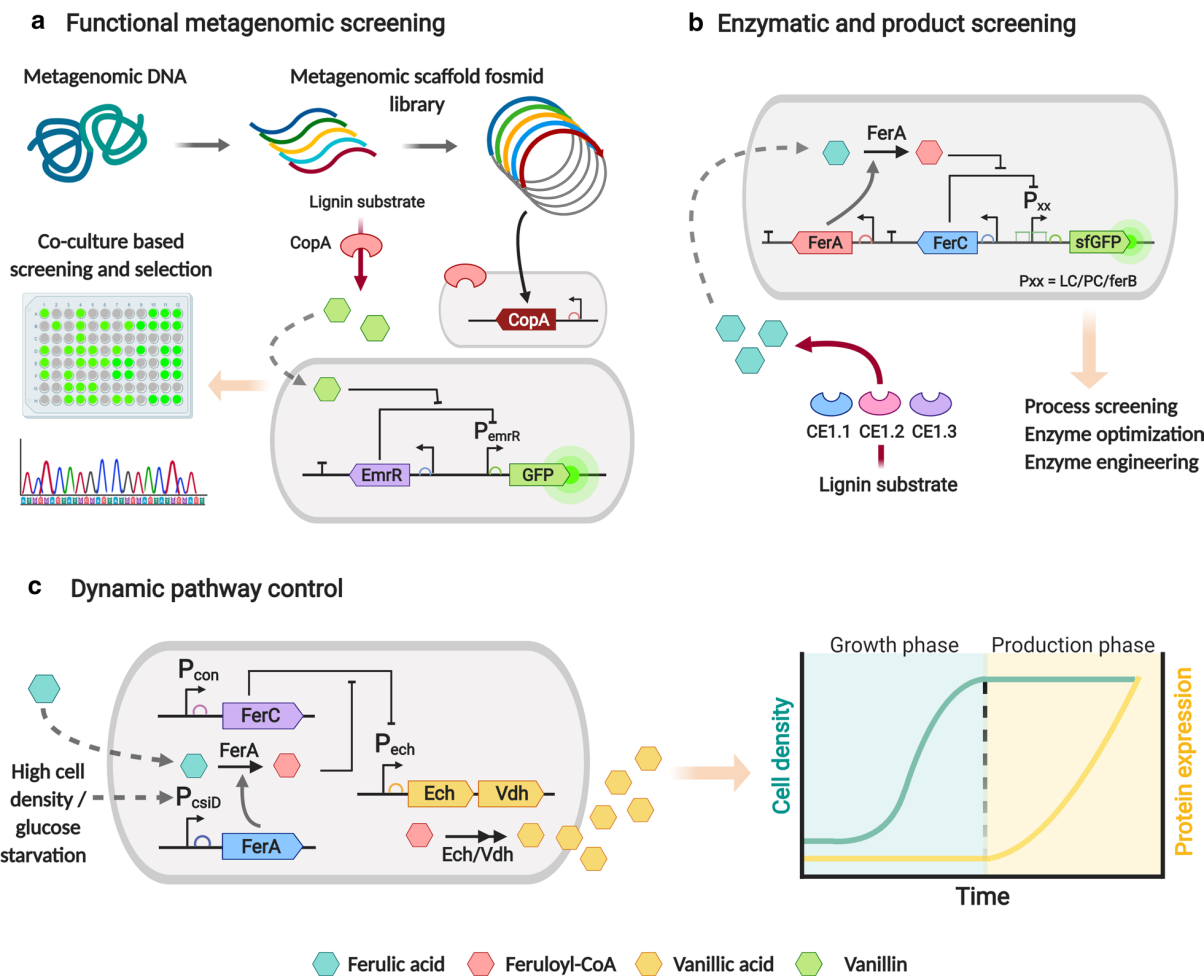
Genetically encoded biosensors and their applications in lignin valorization: **a** functional metagenomic screening method based on an EmrR-based biosensor for identifying lignin-degrading and transforming enzymes from metagenomic samples. A multicopper oxidase (CopA) enzyme capable of degrading lignin to vanillin and syringaldehyde was identified from a recovered metagenomic fosmid DNA library using this system [67]. **b** Transcriptional biosensors as screening methods to allow enzymatic characterization and evolution. A FerC-based biosensor was applied to identify optimal feruloyl esterase (CE1) activity by detecting ferulic acid release [69]. **c** Dynamic pathway regulation system for vanillic acid production. A ferulic acid biosensor was utilized to activate and regulate the biosynthetic pathway only when high cell density was reached and enough substrate was present, diminishing metabolic stress and increasing productivity [68]

High-throughput screens using genetically encoded biosensors allow for the detection and selection of novel biocatalysts from environmental metagenomic samples, which constitute a rich source of genetic information for the discovery of biocatalysts, transporters, and regulatory elements (Fig. 3a) [79, 80]. This approach was applied by Strachan et al. [79], where a predicted multicopper oxidase (CopA) was identified from a community-wide metagenomic scaffold library of bacterial DNA samples isolated from a coal bed, using a genetically encoded biosensor. The biosensor was based on the EmrR aTF and its cognate promoter, which were found to directly respond to mono-aromatic lignin degradation products, including vanillin and vanillic acid (Fig. 3a). The identified enzyme was then employed in the degradation of industrially processed lignin, demonstrating lignin transformation activity [79].

When genetically encoded biosensors are integrated within other screening systems such as FACS, methods can be developed that offer a powerful system for metagenomic sample screening. One such system includes the substrate-induced gene expression (SIGEX) system, a high-throughput promoter-trap method can that can activate expression of reporter genes upon target aromatic induction, and subsequently uses FACS to isolate clones from metagenomic DNA libraries [81]. SIGEX has been employed to identify several catabolic genes and transcription factors that regulate reporter gene expression in response to aromatic compounds, including salicylate and several catechol-derived chemicals such as 4-chlorocatechol [82]. The SIGEX system was also employed by Meier et al. [83], where several aromatic hydrocarbon degrading enzymes and their respective regulators were discovered from aromatic hydrocarbon-contaminated soil samples. The technique subsequently aided the discovery and mapping of operons involved in aromatic hydrocarbon metabolism [83].

### Enzyme, pathway, and strain production screening

Determination of substrate and product concentrations is a major bottleneck for enzymatic optimization and directed evolution processes (see “The screening bottleneck—metabolic pathway engineering and optimization” section). Genetically encoded biosensors have been applied as screening tools to identify active and optimal enzymes and biocatalytic pathways regarding lignin-degrading and transforming enzymes (Fig. 3b) [33, 44, 84]. For instance, the developed FerC repressor-based biosensor described earlier was applied to screen for optimal feruloyl esterase enzyme (CE1) activity against different biomass sources by detecting ferulic acid release (Fig. 3b) [66]. Moreover, the optimized PCA-responsive biosensor based on the pcaU transcription factor from *Acinetobacter* sp. ADP1 was used in the enzymatic screening and selection of dehydroshikimate dehydratase enzymes (AsbF) with PCA-producing activity. Using FACS, individual biosensor cells carrying the desired AsbF could be isolated from an inactive AsbF population [69]. van Sint Fiet et al. [85] utilized a mutated variant of the aTF NahR from *Pseudomonas* to detect benzoic acid and 2-hydroxybenzoic acid and regulate expression of *lacZ* and the tetracycline resistance gene *tetA*. The system was successful in the selection of biocatalytically active cells from an enzymatic library encoding a benzaldehyde dehydrogenase (xylC), as these active mutants allowed benzaldehyde-dependant cell growth [85].

Applying biosensors for enzymatic screening can also facilitate the optimization of productivity of aromatic-transforming enzymes and pathways. Recently, Jha et al. [86] developed a 4HB biosensor in *P. putida* to optimize enzymatic activity and alleviate product inhibition in chorismate pyruvate-lyase (UbiC). Along with the engineered 4HB biosensor–enzyme coupling, benzoic acid was utilized as a product surrogate that inhibited UbiC activity. As a result, they generated an UbiC variant library with reduced product inhibition and isolated a clone that eventually allowed a twofold yield increase in the bioproduction of muconic acid from glucose. Moreover, the *p*-coumaric acid-responsive biosensor developed by Siedler et al. [48] based on the PadR repressor was used in the screening of yeast *p*-coumaric acid production after co-encapsulation within microfluidic droplets. This system was subsequently used in the screening and sorting of high-producing strains from a yeast producer mutant library, indicating that the system could be used to enrich *p*-coumaric acid producing strains [48].

### Dynamic pathway control

Several biosensors have also been developed and integrated into biosynthetic pathways for metabolic engineering applications [41, 87]. Balanced utilization of cellular resources for enhanced chemical production from lignocellulose has been accomplished through the use of biosensors that dynamically regulate intermediate metabolic fluxes and synthetic pathways. Dynamic regulation permits cells to adapt to environmental changes and regulate timely synthesis at each step in a pathway and improve resource utilisation efficiency and productivity (Fig. 3c).

Lo et al. [88] achieved the decoupling of cell growth from metabolite production by dynamically upregulating enzymatic pathways upon nutrient depletion using a biosensor module. The two-layered system allowed separation of the growth and production phases, and selective utilization of target substrates once the growth nutrients had been depleted (Fig. 3c). By applying this system with a *p*-coumaric acid sensing module, metabolic stress was lowered by twofold, while bioproductivity was improved fivefold [88].

Furthermore, an autoregulatory lignin valorization system was developed to diminish aromatic toxicity and circumvent required addition of exogenous inducers [89]. The system was based on the ADH7 vanillin-inducible promoter, which dynamically regulated a catechol biosynthesis pathway only in the presence of vanillin, alleviating metabolic burden and reducing toxicity.

## Other responsive elements available

### Sugar responsive elements

Besides utilizing genetically encoded biosensors to respond to lignin degradation products and intermediates, it is important to develop and implement regulatory systems that can respond to sugars derived from lignocellulose to establish a fully integrated biorefinery process. Hemicelluloses in lignocellulose comprise a very heterogeneous group of polysaccharides, including xylans, arabinoxylans, and cellulose [12]. The hydrolysis and saccharification of these polysaccharides result in the release of sugars, such as xylose, arabinose, and glucose. Constructing biosensors that respond to sugars other than cellulose could help to discover and improve the activity of hydrolysing enzymes towards the development of sugar-fermentative strains for biomass-derived high value production and in second-generation bioethanol production. In addition, these sugars constitute an ideal source of inducers that could be utilized in the dynamic regulation of phenolic and aromatic degrading enzymes for their upgrading into high value chemicals and pharmaceuticals.

To date, several regulation systems have been developed that focus on the detection of these lignocellulose-derived sugars (Table 1). Transcriptional biosensors have been constructed for xylose, cellobiose, and d-arabinose based on the naturally occurring XylR and CelR and on an engineered AraC transcription factor, respectively [90–92]. The latter was constructed by engineering the AraC aTF through random mutagenesis and FACS-based screening to select novel ligand-binding specificity towards d-arabinose [90]. FRET biosensors have also been designed and optimized for the detection of d-arabinose, glucose, and glutamate, using the AraF, MglB, and YbeJ bacterial periplasmic-binding proteins (PBPs), respectively [93, 94]. Moreover, Ribeiro et al. [95] developed a xylose biosensor based on the xylose transcriptional activator XylR and the xylose-binding protein (XBP). The integration of XBP increased the activity of the P_xylF_ promoter by 40%, resulting in increased GFP signal output in the presence of xylose.

These sugar-inducible regulatory systems constitute valuable mechanisms that could be utilized for the simultaneous degradation and biotransformation of hemicellulose and lignin fractions while maintaining reduced product inhibition and increased productivity. Furthermore, the scope of sugars and their analogues that can be detected using biosensors should also be extended. Kelly et al. [96] developed a biosensor based on the l-rhamnose inducible rhaBAD expression system to identify alternative orthogonal effectors. Among these, they found that the sugar analogue l-mannose provided the greatest response fold change [96].

## Conclusion and perspectives

Owing to their importance in the chemical, energy, and pharmaceutical industries, lignocellulosic feedstocks continue to be an attractive source of aromatic compounds. To this end, biomass-derived bioproduction has been modest, achieving low productivity when compared to commercial-scale demands [21]. Effective lignocellulose-derived high value chemical production will likely be accomplished through the discovery and directed evolution of novel biosynthetic pathways and biocatalysts. Emerging biosensor tools for the rapid evolution and high-throughput screening of optimal strains are powerful tools to increase the rate of development and optimisation of such pathways [43]. Biosensor-aided discovery of novel lignin-degrading enzymes will continue to be a crucial tool for the effective detection of lignin degradation capabilities against a wide range of metabolites.

The application of these biosensors is particularly interesting for sampling environmental metagenomes [79], as these constitute a rich and untapped source of enzymatic diversity from uncultivable microbes [97]. Moreover, despite their importance in aromatic transport and metabolic balance, many aromatic transporters and their functions are still unknown or poorly characterized. Biosensors could thus be applied in the identification, validation, and evolution of new substrate-specific transporter proteins [25, 98, 99].

Furthermore, bioproduction of aromatics will need to be incorporated with regulated synthetic pathways to achieve fully integrated dynamic control and production of metabolites to achieve an overall improved bioproduction process. Dynamic pathway control mediated by sugar or aromatic-based regulatory systems will provide a beneficial bioproduction approach by allowing on-demand expression of lignocellulose-transforming biosynthetic pathways when they are most required. To accelerate these biosensor-mediated processes, the current selection of products and intermediate metabolites that can be detected will need to be expanded. Development of novel genetically encoded biosensors will be aided by developments in protein engineering techniques and computational design to engineer novel ligand specificities [42]. In addition, the knowledge gained from advancements in this area can also translate into the development of biosensors with specificity towards complex aromatic molecules, including flavonoids and vitamins [100].

Going forward, the increased adoption of evolutionary strategies is anticipated to aid chemical bioproduction and metabolic engineering developments. Overcoming these current productivity limitations will offer great opportunities for the use of genetically encoded biosensors in pathway engineering for lignocellulose valorization. Advancements in these areas will help diversify the array of products that can be obtained from lignin and provide efficient biocatalysts for their integration into economically viable biorefinery processes.

## Data Availability

Not applicable.

## Abbreviations

BTX: benzene, toluene, xylene
FACS: fluorescence-activated cell sorting
HK: histidine kinases
PBP: periplasmic-binding protein
RR: response regulator
SIGEX: substrate-induced gene expression
